# Viral Respiratory Tract Pathogens During the COVID-19 Pandemic

**DOI:** 10.5152/eurasianjmed.2021.20459

**Published:** 2021-06

**Authors:** Özgür Çelebi, Demet Çelebi

**Affiliations:** 1 Department of Medical Microbiology, Atatürk University Faculty of Medicine, Erzurum Turkey; 2 Department of Medical Microbiology, Atatürk University Faculty of Veterinary Medicine, Erzurum, Turkey

**Keywords:** Pandemia, Covid 19, Viral Respiratory Tract Pathogens

## Abstract

**Objective:**

This study aimed to report viral respiratory pathogens during the coronavirus disease 2019 (COVID-19) pandemic.

**Materials and Methods:**

Other viral pathogens were identified. COVID-19 immunoglobulin M and immunoglobulin G were detected.

**Results:**

Of the 56 samples collected from women, 2 (3.5%) were positive for severe acute respiratory syndrome coronavirus 2 (SARS-CoV-2), whereas 8 (10%) of the 80 samples from men were positive for SARS-CoV-2. The number of respiratory syncytial virus-A–positive cases was 6 (10.7%) in women and 14 (17.5%) in men. Two (3.5%) of the women were positive for parainfluenza-3, and 6 of the men were positive for influenza-B. The number of human metapneumovirus (HMPV)–positive women and men was 6 (10.7%) and 6 (7.5%), respectively. Rhinovirus caused 14.2% and 10% of the cases in men and women, respectively. With a ratio of 10.7% in women and 7.5% in men; SARS-CoV-2, with a ratio of 10% in men and 3.5% in women; influenza-B, with a ratio of 7.5% in men; and parainfluenza-3 and 4, with a ratio of 3.5% in women. SARS-CoV-2 had a mean incidence rate of 7% in men and women. The antibody screening results reveal that antibody formation did not occur in 3 women among the 10 patients who were diagnosed with COVID-19, and antibody formation occurred in 2 of 7 men. Antibody formation occurred in 5 women (16.6%) and 7 men (20.5%) among the 58 patients who were positive for other respiratory tract pathogens. However, 23 (29.5%) of the blood samples collected from 78 individuals who were negative for the COVID-19 agent and other respiratory tract viral pathogens were positive for the COVID-19 antibody.

**Conclusion:**

Because the climate is colder than normal in areas settled at higher altitudes, more than one pathogens act together. In addition, respiratory infections are seen in all seasons. This causes the diseases to be fewer and milder than in other regions.

## Introduction

Respiratory tract infections (RTIs) threaten human health in every stage of life, although they occur more frequently in childhood. Childhood respiratory infections include nasopharyngitis, tonsillopharyngitis, acute rhinosinusitis, acute otitis media, and croup. Both viral and bacterial agents can cause these infections.^1^–^3^ The agent of tonsillopharyngitis in children under the age of 3 years is a virus, whereas the infection is caused by Group A Streptococcus in children between the ages of 4 and 15 years.^2^

Sinus inflammation is observed during the clinical progression of upper RTIs (URTIs), and acute sinusitis can heal spontaneously. The development of sinusitis is facilitated and becomes chronic if underlying problems, such as structural abnormalities of the nose, allergic rhinitis, and immunodeficiency, are present. *Streptococcus pneumoniae* and non-typeable *Haemophilus influenzae* are the leading agents of sinusitis.^4^ URTI is the most frequent cause of admissions to hospitals worldwide, notably in regions with high altitude. The mortality rate in children in the 0–14 age group is 1.2% in Turkey.^5^–^7^

Deaths caused by RTI in adults and children rank fourth worldwide. Viruses, bacteria, and parasites are responsible for RTIs, and viruses cause of about 20% to 60% of these infections.^8^–^10^ Compared with bacterial agents, viral agents cause higher mortality and morbidity and are the primary public health issue. Mortality rates are especially high in developing countries, and it has been claimed that more than 5 million children under the age of 5 years die from RTIs.^9^,^11^ In contrast, the conditions have reversed in the current pandemic, and mortality rates are higher among older people and in developed countries.^12^–^14^

Lower RTIs (LRTIs) are the main culprits of mortality and morbidity in children between the ages of 0 and 3 years^15^, and their agents are mostly viruses. The viruses isolated from LRTIs of children between the ages of 0 and 2 years are respiratory syncytial virus (RSV), rhinovirus, coronavirus, and bocavirus.^16^–^18^

Viruses enter the human body through respiration, colonize in the body, and show systemic spread. Adeno- and rhinoviruses are the causes of cold in childhood, whereas rhino- and coronaviruses are the agents of cold in adults. Together with influenza viruses, adenoviruses are the agents of tracheobronchitis and pneumonia. Adenoviruses are responsible for 5% of acute respiratory infections in children and a lower rate of acute respiratory infections in adults and infect people by colonizing in the small intestine. Coronaviruses also show a similar picture to that of infections caused by adenoviruses in the respiratory tract and intestinal region.^19^,^20^

After the 2000s, severe acute respiratory syndrome coronavirus (SARS-CoV) and Middle East respiratory syndrome coronavirus (MERS-CoV), new types of human coronavirus, emerged as respiratory tract agents in addition to more traditional respiratory viruses, such as RSV, influenza A/B, human rhinovirus, adenovirus, and parainfluenza virus.^21^ Today, in addition to these viruses, SARS-CoV-2 has emerged and caused a pandemic, killing tens of thousands of people.^22^,^23^

RTIs vary depending on age, climate, and seasons, and the progression of their clinical signs ranges from mild to severe. The fact that the agents comprise numerous bacteria and tens of different virus genera cause difficulties and confusion in diagnosis and treatment. These viruses have emerged as the agents of many diseases that turned into pandemics and caused mass deaths in the history of humankind. The agent of the last pandemic is from the coronavirus family. Erzurum is located in the east of Turkey and has a high altitude and cold climate. It snows even in June. Therefore, RTIs such as seasonal flu and cold occur in every season. We aimed to separate the coronavirus disease 2019 (COVID-19) infection, which is the cause of the recent pandemic (March 11–May 15, 2020), from other RTIs and investigate them together considering the lower frequency and severity of the cases in our region compared with the cases in Istanbul and almost non-existent number of cases compared with the cases in Europe.

## Materials and Methods

The first cases of COVID-19 were detected in China, and after the outbreak was declared a pandemic by the World Health Organization, the anxiety surrounding the world was also felt in our region. This study includes 136 cases of individuals between the ages of 0 and 90 years. Throat and nose swabs and blood samples were collected from the patients with the permission (B.30.2.ATA.0.01.00/206-2020) of the ethics committee of the Ataturk University. Animal studies were conducted in accordance with NIH guidelines.

We reached the nasopharyngeal region with the swab sticks by entering from the nostrils. The sticks were turned 360 degrees and swabbed on the tonsils. Then, the swab sticks were placed in transport medium–containing capped tubes (Virocult, Medical Wire & Equipment, Corsham, United Kingdom) and brought to the medical microbiology laboratory that was previously determined as the reference laboratory. Simultaneously with the collection of the swab samples, blood samples were collected into EDTA-containing tubes and brought to the microbiology laboratory. The real-time polymerase chain reaction (RT-PCR) (Model, Bio-Rad, Hercules, CA) method was used for RNA detection in the swab samples, and the nucleic acids of other viral pathogens were identified by referring to the Nucleic Acid Test Quick Reference Guide (20-005-024 and 20-012-024). PCR preparations were made according to the Primer Design 2019-nCoV RT Kit (Bio-Speedy^®^) kit protocol according to the following steps: Oasig OneStep 2X RT-qPCR Master Mix (10 μL), COVID-19 (genesing) and internal control primer probe (2 μL), and RNA (8 μL) was prepared in 1 tube for each patient. Negative and positive controls were included in the study. A Rotorgene Q 5 Plex (Qiagen, Hilden, Germany) RT-PCR device was operated following kit procedure. After separating plasma from the EDTA-containing blood samples, antibody detection was carried out for the agent of COVID-19. COVID-19 IgM/IgG Antibody Rapid Test (Colloidal Gold) kits (Beijing Hotgen Biotech Co. Ltd., Shanghai, China) were used for antibody detection.

## Results

Of 136 individuals, 56 were female and 80 were male. The distribution of the cases by sex is given in Table 1. Table 2 shows the PCR results and rapid test antibody results for the COVID-19 agent and PCR results for other viral agents causing infections in the respiratory tract. Table 3 shows the distribution of these results by sex. Rhinovirus and parainfluenza 3 were positive in 2 of the samples collected from women.

### Statistical Analysis

All data were analyzed by using the chi-square test and SPSS 22.0 (IBM SPSS Corp.; Armonk, NY, USA).

## Discussion

As identified by the World Health Organization, viral diseases have caused severe pandemics and led to mass death for many years. In the last 11 years, they have threatened the entire world and caused major public health issues. SARS-CoV emerged in 2002–2003, H1N1 influenza emerged in 2009, and MERS-CoV emerged in 2012 and posed threats worldwide.^24^ Today (2019–2020), the pandemic across the world continues its effect and causes high rates of mortality and morbidity worldwide; its agent is SARS-CoV-2, which is also among these viruses.^25^,^26^

Their relatively more difficult and expensive identification and propagation compared with microorganisms such as parasites, fungi, and bacteria add to the importance and severity of viral infections. Developing countries such as Turkey and underdeveloped countries are more affected by these infections than developed countries.^27^,^28^

Some of the many viral agents, such as SARS-CoV-2, SARS-CoV, MERS-CoV, influenza species, rhinovirus, RSV, adenovirus, and human metapneumovirus (HMPV), occasionally cause devastating and fatal epidemics and pandemics, whereas some emerge as outbreaks and sporadic cases. The clinical manifestations of RTIs are similar to each other, thus complicating basing their diagnosis on physical findings. The simultaneous emergence and progression of a pandemic caused by a virus, leading to severe clinical manifestation and deaths in groups, and an infection whose manifestation is mild and even unapparent and sometimes emerges only as sporadic cases in the same public are frightening and dreadful in addition to causing diagnostic chaos.^18^,^19^

The results of our study showed that 2 (3.5%) of the 56 samples collected from women were positive for SARS-CoV-2, whereas 8 (10%) of the 80 samples from men were positive for SARS-Cov-2. The number of RSV-A–positive cases was 6 (10.7%) in women and 14 (17.5%) in men. Two (3.5%) of the women were positive for parainfluenza-3, and 6 of the men were positive for influenza-B. The number of HMPV-positive women and men was 6 (10.7%) and 6 (7.5%), respectively. This indicates that the complex picture presented by the simultaneous emergence of multiple virus agents in the same region and population can add to the fear and complicate diagnosis even further. There is a silver lining to this fear and diagnostic difficulty. As we claimed in our previous study, the number of cases of diseases such as COVID-19 is very low, and the diseases progress in a milder fashion with little to no deaths. This likely occurs because of the coemergence of multiple agents in a region, such as Erzurum, that is established on a higher altitude than the average altitude worldwide, and the long and cold winters and cool summers of the region cause RTIs in every season.

In the tested samples, the most frequently detected viral pathogens were RSV-A, causing 17.5% and 10.7% of the cases in men and women, respectively, and rhinovirus, causing 14.2% and 10% of the cases in men and women, respectively. These were followed by HMPV, with a ratio of 10.7% in women and 7.5% in men; SARS-CoV-2, with a ratio of 10% in men and 3.5% in women; influenza-B, with a ratio of 7.5% in men; and parainfluenza-3 and 4, with a ratio of 3.5% in women (Table 4). The totality of these results revealed that as high as 60% of the population in the region were infected by a respiratory tract viral pathogen. SARS-CoV-2 had a mean incidence rate of 7% in men and women. However, its physical and psychological share is close to 100%, thus overshadowing the effect of and fear from other pathogens.^29^,^30^

The antibody screening results in Table 5 reveal that antibody formation did not occur in 3 women among 10 patients who were diagnosed with COVID-19, and antibody formation occurred in 2 of the 5 men. Antibody formation occurred in 5 women (16.6%) and 7 men (20.5%) among the 58 patients (24 women and 34 men) who were positive for other respiratory tract pathogens. However, 23 (29.5%) of the blood samples collected from 78 individuals who were negative for the COVID-19 agent and other respiratory tract viral pathogens were positive for the COVID-19 antibody. The distribution of the positivity in this group by sex was 28.1% for women and 30.4% for men.

Some outcomes of our study and other studies since the first case of COVID-19 was reported in December 2019 include the following:

1. COVID-19 infection is more common in men than in women.
2. Antibody formation against COVID-19 did not significantly differ by sex.
3. Antibody formation in individuals who had mild or unapparent clinical signs was earlier than in individuals who had more severe clinical signs.
4. In individuals who live in regions with high altitudes and were subject to densely colonized viral respiratory tract pathogens, the COVID-19 infection progressed in a milder fashion and deaths were rare.

Although our findings may need revising after more sensitive specific antibody and antigen screening kits are made available, we are of the opinion that our results greatly contribute to the identification of the properties and pathological and sociological effects of the COVID-19 infection and SARS-Cov-2, its agent, on the public.

**Main Points**

- Respiratory tract infections (RTIs) threaten human health in every stage of life, although they occur more frequently in childhood.
- Deaths caused by RTIs in adults and children rank fourth worldwide. Viruses cause of about 20% of these infections.
- Because the climate is colder than normal in areas settled at higher altitudes, more than one pathogen acts together.
- The diseases tend to be fewer and milder than in other regions.

## Figures and Tables

**Table 1:** Ages of the Patients

Age	Women	Men	Total
0–1	8 (28.6%)	20 (71.4%)	28
2–5	12 (35.3%)	22 (64.7%)	34
6–10	6 (75%)	2 (25%)	8
11–20	8 (36.4%)	14 (63.6%)	22
21–50	16 (72.7%)	6 (27.3%)	22
50+ 6	(27.3%)	16 (72.7%)	22
Total	56 (41.2%)	80 (58.8%)	136

**Table 2:** Nucleocapsid and Antibody Results Obtained Using PCR

	Positive	Negative	Total
COVID-19 (with PCR)	10 (7%)	126 (93%)	136
COVID-19 (antibody)	40 (29%)	96 (71%)	136
Other viral respiratory pathogens (with PCR)	58 (46%)	82 (60%)	136

COVID-19, coronavirus disease 2019; PCR, polymerase chain reaction.

**Table 3:** Distribution of the Results by Sex

	COVID-19 (PCR)	COVID-19 (Antibody)	Other viral respiratory pathogens	Total
Female	2 (3.5%)	12 (21.4%)	24 (42.8%)	56
Male	8 (10%)	28 (35%)	34 (42.5%)	80
Total	10 (7%)	40 (29%)	58 (42.6%)	136

COVID-19, coronavirus disease 2019; PCR, polymerase chain reaction

**Table 4:** Distribution of the Viral Respiratory Tract Pathogens by Sex

	Female		Male		Total
	
	(+)	(−)	(+)	(−)	
RSV-A	6 (10.7%)	50 (89.3%)	14 (17.5%)	66 (82.5%)	136
RSV-B	–	56	—	80	136
RhinoV	8 (14.2%)	48 (85.8%)	8 (10%)	72 (90%)	136
Parainf.1	–	56	–	80	136
Parainf.2	–	56	–	80	136
Parainf.3	2 (3.57%)	54 (86.43%)	–	80	136
Parainf.4 2	(3.57%)	54 (86.43%)	–	80	136
Influ.A	–	56	–	80	136
Influ.AH1	–	56	–	80	136
Influ.AH3	–	56	–	80	136
Influ.B	–	56	6 (7.5%)	74 (92.5%)	136
HMPV	6 (10.7%)	50 (89.3%)	6 (7.5%)	74 (92.5%)	136
Adenovi.	–	56	–	80	136
Total	24 (42.8%)	32 (57.2%)	34 (42.5%)	46 (57.5%)	136

Adenovi., adenovirus; HMPV, human metapneumovirus; Influ., influenza; Parainf., parainfluenza; RhinoV, rhinovirus; RSV, respiratory syncytial virus.

**Table 5:** Antibody Results for All Samples

	Female		Male		Total
	
	(+)	(−)	(+)	(−)	
With PCR (+) (COVID-19)	—	3	2 (28.5%)	5 (71.5%)	10
With PCR (+) (Resp. tra. path.)	5 (16.6%)	19 (84.4%)	7 (20.5%)	27 (89.5%)	58
With PCR (−) (Resp. tra. path.)	9 (28.1%)	23 (71.9%)	14 (30.4%)	32 (69.6%)	78

COVID-19, coronavirus disease 2019; PCR, polymerase chain reaction; Resp. tra. path., respiratory tract pathogen.
